# *Hombre Seguro* (Safe Men): a sexual risk reduction intervention for male clients of female sex workers

**DOI:** 10.1186/1471-2458-14-475

**Published:** 2014-05-20

**Authors:** Eileen V Pitpitan, Claudia V Chavarin, Shirley J Semple, Carlos Magis-Rodriguez, Steffanie A Strathdee, Thomas L Patterson

**Affiliations:** 1 Division of Global Public Health, Department of Medicine, University of California, San Diego, USA; 2 Centro Nacional para la Prevención y Control del VIH/SIDA (CENSIDA), Ministry of Health, Mexico City, Mexico; 3 Department of Psychiatry, University of California, San Diego, USA; 4 Department of Psychiatry, University of California, 9500 Gilman Drive, La Jolla, San Diego, Mail Code 0680, CA 92093-0680, USA

## Abstract

**Background:**

Male clients of female sex workers (FSWs) are at risk of HIV and other sexually transmitted infections (STIs). We conducted a two-arm randomized controlled trial to test the efficacy of a sexual risk reduction intervention for male clients of FSWs in Tijuana, Mexico.

**Methods/Design:**

Male clients of FSWs who were at least 18, were HIV-negative at baseline, and reported recent unprotected sex with FSWs were randomized to the *Hombre Seguro* sexual risk reduction intervention, or a time-attention didactic control condition. Each condition lasted approximately one hour. Participants underwent interviewer-administered surveys and testing for HIV and other STIs at baseline, and at 4, 8, and 12 month follow-ups. Combined HIV/STI incidence and unprotected vaginal and anal sex acts with FSWs were the primary outcomes.

**Discussion:**

A total of 400 participants were randomized to one of the two conditions. Analyses indicated that randomization was successful; there were no significant differences between the participants in the two conditions at baseline. Average follow-up was 84% across both conditions. This is the first study to test the efficacy of a sexual risk reduction intervention for male clients of FSWs using the rigor of a randomized controlled trial.

**Trial registration:**

NCT01280838, Date of registration: January 19, 2011.

## Background

HIV behavioral and intervention development research is necessarily directed at groups that engage in high HIV transmission risk behavior, including female sex workers (FSWs). Whereas there has been much attention focused on the HIV risks of FSWs [1-6], there is a dearth of literature focusing on their male clients. Male clients who have unprotected sex with FSWs and other female partners in the general population create a possible bridge from a high risk to a low risk population, since some are married or have other partners [7,8]. Globally, research has shown that male clients of FSWs engage in risky sexual behaviors [9-13] and have a higher incidence of sexually transmitted infections (STIs) compared to other men [14-19]. While interventions to reduce HIV risks of FSWs have been developed and evaluated [5,20,21], to date, we have not identified an existing intervention designed to reduce sexual risk behavior and HIV and other sexually transmitted infections among male clients of FSWs. Regions affected by a growing HIV epidemic may benefit from such an intervention program. Border-regions in particular, like the U.S.-Mexico border, may especially be in need as this is an area where a dynamic HIV epidemic exists and where border-crossing male clients of FSWs may potentially bridge epidemics across the countries.

San Diego, California and Tijuana, Baja California abut one another along the extreme western end of the U.S.–Mexico border. Together, these two cities form the world’s largest binational metropolis and share its busiest land border crossing [22]. In both cities, FSWs offer sexual services to men from Mexico and the U.S. Estimates of the number of FSWs in Tijuana range from 6000 to 10,000 [23]. Because prostitution is illegal in San Diego, U.S. citizens frequently travel to Tijuana for sex, where it is quasi-legal in the *zona roja* (red light zone). Since ~60% of the U.S. Hispanic population is of Mexican origin, and 42,000 Tijuana residents cross the border to work in San Diego every day [24], health problems affecting the border region are important for both countries. Since border cities in Mexico are economically depressed and commercial sex work is quasi-legal, the U.S.-Mexico border region is a nexus for prostitution, drug abuse and sexual tourism, and rising rates of HIV and STIs.

In June 2012, the estimated number of individuals living with HIV in Mexico was about 179,500, with an HIV prevalence of 0.2% [25]. However, HIV prevalence among adults in Baja California, which includes Tijuana, the state’s major city, was double (0.54%) [26,27]. National and state-level HIV/AIDS surveillance data mask a dynamic HIV epidemic in Tijuana, where HIV prevalence among FSWs has risen from 2% in 2003 to 6% [28]. The evidence suggests that the HIV epidemic has moved from low-level to concentrated, and could become more generalized if these trends continue.

In response to the growing epidemic among FSWs in Tijuana, our research team developed and evaluated the efficacy of a brief behavioral intervention called *Mujer Segura* (Safe Women)*.* The intervention was designed to increase condom negotiation skills and reduce HIV/STI incidence among FSWs in U.S.-Mexican border cities [29]. The team collected data in Tijuana and Ciudad Juarez (which borders El Paso, Texas) from 2004 to 2006. In each city, 450 FSWs aged >18 without known HIV infection who reported unprotected sex with >1 client within the previous 2 months were randomized to an attention-control condition or an intervention integrating motivational interviewing (MI) and theoretical principles of behavior change. The intervention incorporated a core set of interrelated constructs derived from Social Cognitive Theory (SCT) [30,31], and the Theory of Reasoned Action (TRA) [32,33]. Outcome data from Tijuana and Ciudad Juarez, where overall follow-up was 83%, found a 40% reduction in combined HIV/STI incidence and significant reductions in unprotected vaginal and anal sex with clients [5]. These findings demonstrated the feasibility and efficacy of a brief intervention to reduce HIV/STI incidence among FSWs on the U.S.-Mexico border. Despite the moderate effect size, STI incidence remained high in the intervention arm, suggesting that additional strategies, such as targeting male clients of FSWs for interventions, are needed.

Our team conducted the first study of male clients of FSWs in Tijuana, Mexico in 2008 [34]. This pilot work revealed that male clients have a comparable HIV prevalence to FSWs in this region (~5%). Clients reported sex with an FSW an average of 26 times in the past year, and once every two weeks in the past four months. Half reported recent unprotected sex with FSWs, which was associated with drug use. Drug using clients were also more likely to engage in sexual bridging, or unprotected sex with both FSW and their steady partners [8]. Thus, male clients of FSWs in Tijuana are at high risk for HIV and other STIs. An efficacious intervention program to reduce this risk is both timely and imperative.

The protocol herein describes a two-arm randomized control trial called *Hombre Seguro* (Safe Men) to test the efficacy of a sexual risk reduction intervention among male clients of FSWs in Tijuana, Mexico. A total of 400 clients were recruited into the trial. By design, half of the participants were residents of San Diego, and the other half were residents of Tijuana. We hypothesized that compared to participants in the didactic control group, participants in the *Hombre Seguro* intervention group will report less unprotected vaginal and anal intercourse with FSWs and have a lower incidence of HIV and other STIs.

## Methods/Design

The study protocol was submitted, reviewed and approved by Institutional Review Boards in the US (University of California San Diego) and Mexico (Comite de Etica sobre Salud Y Poblacion).

### Study sample

Study recruitment took place between September 2nd, 2010 through October 9th, 2012.

#### Inclusion criteria

Participants were required to be (i) biologically male, (ii) living in either Tijuana or San Diego County, (iii) at least 18 years old, (iv) report having purchased sex for money, drugs, shelter or goods in the last 4 months, (v) test HIV-negative at baseline, and (vi) agree to receive antibiotic treatment for Chlamydia, gonorrhea, and syphilis if they test positive (to allow us to differentiate incident from prevalent cases at follow-up). Additionally, clients must have reported having had unprotected vaginal or anal sex with a FSW in Tijuana at least once during the previous 4 months.

#### Exclusion criteria included

(i) consistent use of condoms for vaginal and anal sex with all FSWs during the previous 4 months; (ii) being <18 years old; (iii) being transgendered or female; and (iv) testing HIV-positive. The age criterion was imposed for the following reasons: it is more difficult for men <18 years old to cross the border; the proportion of men in pilot work who reported having had sex with an FSW before they were 18 was low (7%); and a specialized intervention that takes into account the needs of younger men would be needed. Since younger clients do exist, we referred these men to non-governmental organizations in either San Diego or Tijuana that provide services to younger men. Transgendered men were excluded because they have different relationship dynamics and socio-cultural risk profiles that would warrant a potentially different intervention.

##### Description of field staff

The Tijuana field team was comprised of 1 field coordinator-interviewer, 1 outreach worker, 2 counselors, and 1 nurse. The field coordinator and the outreach worker are both Hispanic males who are well known in the community and are highly familiar with the target population. They performed many tasks, which included identifying, recruiting, tracking, and scheduling participants for baseline and follow-up interviews. They were also responsible for contacting and working with “jaladores,” (men who connect interested clients to FSWs or sex work venues). Jaladores assisted in the identification of US residing clients and provided assistance to locate clients for their follow-up interviews. The two counselors were Hispanic females, one a psychologist and the second a nurse trained to conduct both the control and intervention counseling sessions. The field coordinator and the outreach worker were also trained to apply the surveys and provide the counseling sessions in English and Spanish. The field nurse is a Hispanic female certified as a laboratory technician. She is skilled in the collection and shipment of all biological samples for STI testing, and tracked STI results and treatment referrals. The whole team shared the responsibilities of scheduling participants for their follow-up assessments, performing receptionist duties, and assisting with other office activities.

#### Participant recruitment

We used time-location sampling within each *colonia* (neighborhood). This method was successfully used in our survey and pilot work, and has been employed in other studies to recruit hidden samples of MSM, including Latinos [35,36]. With the help of outreach workers and our community advisory board (CAB), we compiled a map of bars, brothels, shooting galleries, hotels, alleys and street corners. Following Stueve et al [35], we randomly selected three recruitment locations each month from *colonias* assigned to each of the two conditions. A specified number of days and times were randomly selected, and the selected locations were visited during the specified period. Trained outreach workers familiar with these establishments and their clientele approached prospective participants. Using personal digital assistants, workers recorded the number, approximate age, and (where applicable) reason for refusal or ineligibility. Men who appeared eligible and were interested in participating were given a study card and asked to present it when they visit the study office. HIV testing, informed consent, randomization, baseline survey and the intervention occurred in the study office to avoid logistical problems in the FSWs’ workplaces. A leading human rights advocate in Tijuana, and a member of our CAB, assisted us in explaining the study to the bar and hotel owners at a community meeting, as we did in *Mujer Segura.* This ensured that our study staff were met with respect and trust as we began to recruit male clients from these establishments.

As our study progressed, drug related violence waxed and waned. As violence increased, some U.S. clients avoided public places by utilizing jaladores to rent hotel rooms, deliver FSWs, alcohol and drugs. In order to access these men jaladores recruited men as they crossed into Mexico.

#### Participant screening

A five-minute screener was developed to identify and exclude ineligible men. Questions included age, engaging in sex in exchange of money, drugs, goods or shelter and date of last exchange, date of last unprotected vaginal or anal sex act, residence and intention to move in the next year. Also included were a few non-related “red herring” questions (e.g., transportation needs) to prevent men from guessing eligibility criteria. Men deemed potentially eligible underwent the informed consent process and a rapid HIV test. Men who were drunk or too “high” on drugs to provide informed consent were rescheduled.

### Data collection

#### Baseline interview

The baseline interview took approximately 45 minutes to complete and was theory-driven and translated into Spanish and back-translated into English by our bilingual and bicultural staff who reviewed questions for cultural and linguistic appropriateness. All measures were administered using computer-assisted personal interviewing (CAPI; NOVA software, MD, USA).

Socio-demographic and family background variables were queried (e.g., city or town of birth, education, number of children, marital status, religion, living situation). History and practices regarding substance use included age at first use of alcohol and specific drugs; amount of alcohol consumed (using the AUDIT); [37] types of substances used alone and in combination (e.g., heroin, cocaine, crack, methamphetamine, GHB, ketamine, inhalants) and routes of administration; injection use; frequency of injection; syringe cleaning; syringe sharing; and history of drug treatment [38]. Clients were also asked to report their use of alcohol and drugs before or during sex with FSWs in the past 4 months.

Sexual behaviors included number and frequency of unprotected vaginal and anal sex acts with FSWs, with spouse or steady partner, and with casual partners in the past 4 months; number of partners who inject drugs; number of male sex partners; and use of the male or female condoms. Because half of the sample were San Diego residents and half were Tijuana residents, and Tijuana residents may travel to San Diego where they might have sex, sexual risk behavior was assessed in relation to both Tijuana FSWs and San Diego County FSWs.

Contextual factors included sexual venues (e.g., bar, street corner, strip club), availability of condoms, commodity and value exchanged, and context of offering FSWs incentives for unprotected sex acts. Psychosocial factors included lifetime history of abuse (emotional, physical, or sexual) and depressed mood, which was assessed using the 10-item CES-D scales [39]. These measures were used previously in *Mujer Segura* and were deemed culturally appropriate by our binational team.

Psychosexual factors included misogyny, sexual compulsivity, sexual sensation-seeking, social sexual effectiveness, attitudes toward male sexuality, and traditional machismo.

### Mechanisms of change variables

#### HIV knowledge

We utilized a measure of knowledge consisting of 18 items [40], which assessed awareness of the importance of condom use with respect to HIV/STI prevention (e.g., “People who have been infected with HIV quickly show serious signs of illness”). Response categories were True/False.

#### Self-efficacy towards condom use

This 8-item measure was based on a scale we developed for our sex work research in Mexico [29]. Participants were asked to indicate the extent to which they are able to use a condom properly. Responses were coded on a 4-point scale (1 = Strongly Disagree to 4 = Strongly Agree). Example items include, “I can use a condom properly;” and “I can use a condom in any situation (e.g., with different partners or in different places)”.

#### Outcome expectancies

Participants responded to 11 items using a scale ranging from 1 (Strongly Disagree) to 4 (Strongly Agree) that has been used in our sex work research in Tijuana [29]. Sample items are “I will be more relaxed if my partner(s) and I use a condom;” and “I will feel good about myself if I talk about safer sex with my sexual partner(s).”

#### Randomization

After screening was completed, participants were randomly assigned to one of two conditions based on a randomization schedule that was generated *a priori* by the study statistician. The randomization schedule was not disclosed to the interviewers, ensuring that interviewers were blind to group assignment.

#### Overview of intervention and control condition

##### Intervention condition

Male clients randomized to *Hombre Seguro* received the active safer sex intervention, which was developed based on our experience conducting several large-scale sexual risk reduction interventions in the U.S. and Mexico, including *Mujer Segura* and our pilot work. The intervention incorporates principles of MI, SCT, and TRA. Experienced interviewers who are indigenous to these communities were trained to deliver this intervention. The intervention, and control condition, took an average of 60 minutes to deliver. The main steps in the *Hombre Seguro* intervention are delineated below.

*Steps 1 and 2: Determine Readiness for Change and Decisional Balance Exercise:* The counselor began the session by having the participant complete the “ready, willing, and able” exercise. This exercise assesses the participant’s readiness for change, thereby providing valuable information to guide the session. This activity is followed by the decisional balance exercise, which is designed to help the client understand motivations that underlie his sexual risk behavior with FSWs. Using this approach, the counselor asked the client to describe the pros and cons associated with not using condoms with FSWs, in order to facilitate the personal realization that these behaviors entail more negative than positive outcomes.

*Steps 3 and 4: Explore Participant’s Attitudes Toward Condoms and Unsafe Sex: Build Motivation for Change:* Using MI techniques (e.g., key questions, reflective listening, summarization, affirmation), the counselor and participant discussed underlying motivations for risky sexual behavior with FSWs. Through this approach, the client gains insight into his behavior and begins to build motivation for change. This is accomplished by eliciting self-motivated reasons for change and enhancing the participant's self-efficacy for change. Self-motivational statements obtained from male clients in our pilot work include: “I should use condoms to protect myself and my wife” and “The sex will probably last longer if I use a condom with the girls.”

*Steps 5 and 6: Assess High-Risk Situations, Explore Triggers of Unsafe Sex (i.e., Drug Use), and Discuss Coping with Cravings and Urges:* The counselor asks the male client questions about condom use and drug or alcohol use during sex, his perceived need to change, possibility of change, self-efficacy for change, and stated intentions to change, working to increase his awareness of unsafe sex and associated risks with FSWs (e.g., HIV and STIs). A primary goal of the counseling session was to help participants develop insights into motivations and triggers of unsafe sex with FSWs. A range of motivators and personal triggers of unsafe sex were explored (e.g., negative attitudes toward condoms, negative attitudes toward FSWs). In addition, the counselor queried the participant as to what extent his sexual risk behaviors with FSWs are a function of substance use. The counselor discussed the participant's desire and willingness to change his high-risk sexual practices with FSWs. Using a CBT approach, the counselor and participant explored the role that thoughts, feelings, and actions play in changing high-risk sexual behavior. The counselor taught the participant skills for dealing with cravings and urges for risky sex, including reframing thoughts, avoiding or leaving a high risk situation, engaging in a distracting activity, and delaying the decision to seek out a sex worker.

The counselor helped the participant to see possible links between his substance use and high-risk sexual behavior with FSWs. The counselor explored the participant’s past and current use of substances. The participant was asked to generate a list of problems and concerns regarding his drug use. The counselor and participant then problem-solved each issue and the participant then generated target goals for drug use (e.g., stop using drugs, use on weekends only). Through these exercises, male clients were encouraged to develop at least one attainable goal to reduce their injection risks (e.g., avoid being high with FSWs or wait until after sex to use drugs, obtain their own sterile syringe and “works” before using, bleach used syringes).

*Steps 7–9: Problem-solve Barriers to Safer Sex, Knowledge and Skill-Building Exercises, and Safer Sex Role-play:* A list of barriers to safer sex were generated; the counselor and participant worked together to problem-solve each by listing advantages and disadvantages of every solution and weighing and prioritizing alternatives to select the most promising. The participant actively participated in problem-solving and was encouraged to come up with his own solutions. He was presented with a menu of safer sex options ranging from using condoms, having oral instead of unprotected vaginal or anal sex, avoiding sex when high on drugs, waiting to use drugs until after sex, and saying “no” to FSWs who want to perform sex without a condom. Role-plays were used as the primary exercise for problem-solving potential barriers to safer sex with FSWs. According to both MI and SCT, belief in one’s ability to bring about change is an important motivator of change. The counselor helped the client define achievable goals (e.g., always use a condom for vaginal or anal sex with FSWs). Once the participant had defined goals and arrived at a plan of action, the counselor aimed to strengthen the participant’s commitment to using condoms by exploring ways to make condom use more appealing. The counseling session also promoted consistent use of condoms through modifying participants’ thoughts, feelings, and actions with respect to condom use. Social cognitive strategies, which include increasing knowledge, self-efficacy, and positive outcome expectancies in relation to condom use, were utilized [31].

Another step in counseling involved knowledge and skill-building exercises (e.g., condom use demonstration, role modeling). Together, the counselor and participant discussed their successes, and the counselor made suggestions for improvement if necessary. Another goal of the counseling session was to enhance communication and assertiveness skills. Problem-solving barriers to unsafe sex was placed in the context of teaching the participant effective communication skills. Through role-play exercises, male clients practiced effective communication skills so that they felt comfortable turning down any offer for unprotected sex from FSWs.

#### Didactic control condition

The didactic control condition was a modified version of the CDC’s revised guidelines for HIV counseling, testing, and referral [41] and materials from Mexico’s National Center for AIDS Studies (CENSIDA) [42] that was used in *Mujer Segura*. The one-session, 60-minute didactic control counseling condition focused on HIV and STI prevention, risk appraisal, and the development of a risk reduction plan. First, the counselor guided the participant through a personal risk assessment. This involved enhancement of HIV/AIDS knowledge and of the participant’s self-perception of risk. The participant and counselor explored specific recent risk incidents, reviewed previous risk reduction experiences, and explored barriers to change. Counseling topics included enhancement of HIV/AIDS knowledge, synthesis of risk incidents and risk patterns, and the development of a personalized risk reduction plan. In this condition, there were no theory-driven, active skill-building elements oriented towards safer sex practices.

#### Outcome ascertainment

To ascertain HIV status at baseline and follow-up visits, we used the Advanced Quality™ Rapid Anti-HIV (1&2) test which is a colloidal gold-enhanced, immunochromatographic assay for the qualitative detection of HIV antibodies in whole blood, serum or plasma. All reactive samples were then tested at the San Diego County Public Health Laboratory (SDCPHL) using HIV-1, 2 serum antibody enzyme immunoassay (EIA) and indirect fluorescent antibody (IFA) tests. Clients were also screened for syphilis, chlamydia, and gonorrhea. Syphilis serology included a rapid diagnostic screening for the qualitative detection of antibodies to *Treponema pallidum* in serum. All reactive samples were subjected to the rapid plasma reagin (RPR) test and the *T. pallidum* particle agglutination assay (TPPA). Testing for Chlamydia and Gonorrhea was conducted using the APTIMA COMBO 2 Assay which is a target amplification nucleic acid probe test that utilizes target capture for the in vitro qualitative detection and differentiation of ribosomal RNA (rRNA) from *Chlamydia trachomatis* (CT) and/or *Neisseria gonorrhoeae* (GC) from urine samples using the TIGRIS DTS Automated Analyzer or semi-automated instrumentation as specified. At baseline and follow-up visits, blood specimens were obtained by venipuncture and centrifuged on site. Urine samples were collected and transferred to Gen-Probe Aptima Combo 2 Assay tubes for *C. trachomatis* and *N. gonorrhoeae*. Specimens were labeled with the participant’s unique study identification number, date of birth and collection date. Serum and urine samples were batched and stored at −20 degrees Celsius in on-site freezers until their transport to San Diego on a weekly basis.

#### Biological sample transport

Biological samples were imported from Tijuana to SDCPHL for confirmatory testing. Transport of samples constituted a binational effort with the SDCPHL, the State Health Secretary of Baja California and UCSD. A US Centers for Disease Control and Prevention (CDC) permit for the importation of biological samples was secured for the duration of the study and samples were prepared following municipal, state and international standards that regulate the handling, packing, transporting and delivery of biological specimens in Mexico and the U.S. Cross-border transport of samples followed a weekly schedule and was facilitated by a customs broker in Mexico and our transporters holding a Secure Electronic Network for Travelers Rapid Inspection (SENTRI)-card issued by the U.S. Department of Homeland Security’s Customs Border Protection Department.

#### Pre- and post-test counseling and referrals

After each interview at baseline and follow-up, post-test counseling for HIV/STI testing were performed as per CDC guidelines, which are compatible with those from the Mexico Ministry of Health. Follow-up visits to provide STI test results occurred when results were available, approximately one to two weeks after testing.

Any Mexican citizens who tested positive for HIV were referred for treatment to the CAPASITS Clinic where free care and anti-retrovirals are provided under Mexico’s national health care system. Any U.S. residents who tested positive for HIV were referred for treatment to the San Diego County Department of Health. Those testing positive for syphilis, gonorrhea or Chlamydia received free treatment offered by the CAPASITS clinic in Tijuana or by the San Diego County Department of Health. We ensured that all men received STI treatment regardless of their randomization assignment, at both baseline and follow-up. At each visit, men were counseled to avoid self-medicating STI symptoms to avoid promoting resistance. To encourage men to return for STI treatment, they were given a taxi voucher not to exceed $20 USD per visit. Our CAB felt that these items were preferable to cash incentives to avoid setting a precedent that the community cannot sustain. Men were also encouraged to refer their sex partners (including FSWs) for free STI testing and treatment at the Municipal Health Clinic in Tijuana or at the San Diego Department of Health.

### Participant incentives

Enrolled participants received reimbursements ranging from $5 to $25 US dollars upon completion of specific study activities as follows: a) Five dollars was provided for completing study screening; b) Twenty dollars was provided upon completion of the baseline interview; c) Ten dollars was provided for completing the control, or intervention counseling session; d) Twenty-five dollars was provided upon completion of quarterly follow-up visit.

#### Follow-up interviews

Follow-up interviews were conducted at 4-, 8-, and 12-month post-randomization and were interviewer administered using CAPI. At follow-up participants were re-tested for HIV and STIs and underwent follow-up interviews with recall periods that referred to the period since the last interview.

#### Cohort retention

Our research experiences in Mexico through the years has led to improved retention methods. These include phone and pager reminder calls, tracing through self-reported contacts, monetary reimbursement, and small tokens (e.g., toiletries). Reimbursements of $25 USD have been used previously in our settings and have reduced attrition elsewhere^121^. We conducted street tracking, and posted notices at shelters, clinics and drug treatment programs on both sides of the border. Since mail is not reliable in Mexico, we tracked participants by asking them to show on a map where they live (e.g., vacant lots, parks, canyons) and work (e.g., hotels, bars). We obtained IRB approval at UCSD and in Mexico to allow follow-up of subjects who became incarcerated or hospitalized. Data were maintained and updated in a database that was password protected and separated from interview data. Each month, the data manager printed out a list of those due for an appointment. Passive follow-up included seeking permission from IRBs to link to available jail, prison, hospital and INS rosters in a confidential manner. Semi-annually, we searched the “Registro Civil,” where death certificates may be accessed publicly.

#### Fidelity

We employed the following measures to ensure the fidelity of our intervention: First, all counselors underwent intensive training. Second, a random 10% of counseling sessions were audio recorded with the participant’s permission and were reviewed by the project director and scored for fidelity to the intervention messages. Immediate corrective action and re-training was undertaken when necessary.

#### Contamination

There were at least two potential sources of contamination between intervention conditions: (i) our counselors and (ii) cross talk between men assigned to different conditions. We chose cluster randomization over individual randomization to minimize contamination. Rigorous training and fidelity checks described above also helped guard against contamination introduced by our counselors. We found that between-participant contamination is rare (e.g., <1% in *Mujer Segura*). Nevertheless, at follow-up, we asked men if they had talked about safer sex with another male client, and if so, what the conversation included. This information can be used as a covariate in our analyses. Follow-up interviews also asked men if they have self-medicated with antibiotics that may mask detection of incident STIs, which we can also take into account in our analyses. Regardless of randomization assignment, men were encouraged to return for treatment referral to our project offices in either Tijuana or San Diego any time they experience STI symptoms. Such cases will be recorded as STI events, which are one of our study outcomes.

#### Power calculations

We used the software program PASS (NCSS, Kaysville, UT) to calculate power. A 20% attrition rate was assumed based on our previous research experiences (*i.e.*, N = 400*0.80 = 320). All power calculations were based on two-sided tests, with a 0.05 type I error rate. Each calculation took into account clustering.

*Power to detect an intervention effect on unprotected vaginal and anal sex:* Power calculations were based on a two-sided Poisson Regression of a dependent variable of counts (*i.e.*, number of unprotected sex acts) on a binary independent variable (Group1 = active risk reduction, Group2 = didactic presentation) with proportion of 0.50 (*i.e.*, 160 participants in each treatment group). We found that if the 1-year rate of unprotected to total number of sex acts in the didactic presentation group is 35%, 40%, 45%, or 50%, we will have at least 80% power to detect a rate ratio (Group1/Group2) of at least 0.59 (*i.e.*, 21% rate of unprotected to total sex in the active risk reduction group), 0.61 (24% in the active risk reduction group), 0.63 (28% in the active risk reduction group), and 0.65 (33% in the active risk reduction group), respectively.

*Power to detect an intervention effect on HIV/STI incidence:* Power calculations were based on a two-sided Poisson regression of a dependent variable of counts (*i.e.*, number of new STIs) on a binary independent variable (Group1 = active risk reduction, Group2 = didactic presentation) with proportion of 0.50 (*i.e.*, equal number of participants in each group). We found that if the 1-year combined HIV/STI incidence rate for the didactic presentation group is 10%, 15%, 20%, or 25% we will have at least 80% power to detect a rate ratio (clients in active risk reduction group vs. clients in the didactic presentation group) of at least 0.35, 0.44, 0.49, and 0.53, respectively.

## Results

**Figure 1:**
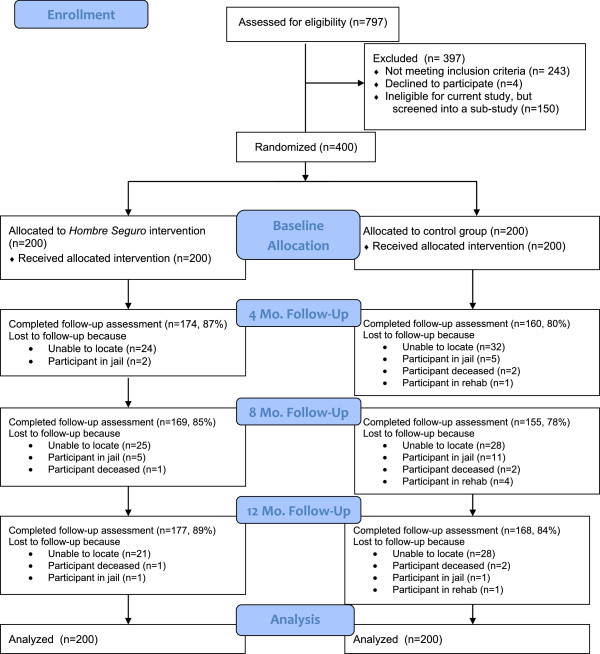
Consort diagram summarizing participant flow through the study.

Figure 1 displays the CONSORT diagram. A total of 400 male clients of FSWs were recruited into the study, 197 from San Diego, 203 from Tijuana. A total of 797 men were screened for participation, of whom a total of 397 were excluded. There were 243 (30.5%) men who were ineligible due to inclusion/exclusion criteria: 191 (78.6%) did not report unprotected vaginal or anal sex with a FSW in Tijuana in the past four months, 12 (4.9%) did not plan to reside in Tijuana or San Diego County for the study period (12 months), 12 (4.9%) tested HIV-positive, 10 (4.1%) were not a resident of Tijuana or San Diego County, 9 (3.7%) reported being told by a health professional that he was HIV-positive, 4 (1.6%) had steady relationships with only FSWs in the past four months, and 1 (<1%) was less than 18 years of age. There were 4 men (1.6%) who refused to participate. The remaining 150 men were ineligible for the current study but were screened for recruitment into a sub-study of *Hombre Seguro.*

Of the 400 eligible men, 400 provided informed consent, completed the baseline interview, and provided biological samples. At the same visit 200 men were randomized into the *Hombre Seguro* intervention and 200 into the control group. Table 1 provides information about the socio-demographics and baseline characteristics of the 400 participants, overall and by group assignment. Overall, there were no statistically significant differences between the two groups, supporting randomization as successful. The only marginally significant difference that was found was for having any children, with participants in the control group trending to be more likely to have children than participants in the intervention group (p = .052). Both groups were balanced with respect to socio-demographics and baseline behavioral and STI characteristics.Follow-up rates were excellent, as shown in Figure 1, the average follow-up was 83.8% across both conditions. The main reasons for being lost to follow-up were that the participant was unable to be found, jailed, deceased, or in rehab.

**Table 1:** Descriptive statistics of Hombre Seguro participants by intervention group at baseline (n = 400)

**Variable**	**Total (n = 400)**	***Hombre Seguro *** **(n = 200)**	**Didactic Control (n = 200)**	p**
***Sociodemographics***				
Age (years)	37 (30, 45)	37 (30, 44)	38 (30, 45.75)	.26
Hispanic (v. non-Hispanic)	350 (87.5%)	174 (87.0%)	176 (88.0%)	.76
U.S.-born	82 (20.5%)	43 (21.5%)	39 (19.5%)	.62
Live in U.S. (v. Mexico)	197 (49.3%)	100 (50.0%)	97 (48.5%)	.76
Ever deported from U.S.	136 (34.0%)	71 (35.5%)	65 (32.5%)	.53
Married/common law	126 (31.5%)	60 (30.0%)	66 (33.0%)	.52
Has a wife, steady partner, or girlfriend	186 (46.5%)	93 (46.5%)	93 (46.5%)	1.00
Resides with spouse or steady partner	108 (27.0%)	52 (26.0%)	56 (28.0%)	.65
# of years of education	9 (7, 12)	9 (6, 12)	9 (7, 12)	.69
Has children	276 (69.0%)	129 (64.5%)	147 (73.5%)	.05
Employed	250 (62.5%)	131 (65.5%)	119 (59.5%)	.22
Annual income	18 K (8 K, 30 K)	19 K (9 K, 35 K)	18 K (8 K, 27 K)	.48
Bisexual (v. Heterosexual)	44 (11.0%)	22 (11.0%)	22 (11.0%)	1.00
Jailed*	77 (19.3%)	38 (19.0%)	39 (19.5%)	.90
Parole*	19 (4.8%)	11 (5.5%)	8 (4.0%)	.48
***Alcohol and Drug Risks***				
AUDIT score ≥ 8	197 (49.3%)	103 (51.5%)	94 (47.0%)	.37
Ever used any illicit drug	373 (93.3%)	183 (91.5%)	190 (95.0%)	.16
Recently used any illicit drug*	321 (80.3%)	158 (79.0%	163 (81.5%)	.88
***Sexual Risks***				
Drunk during sex with a FSW*				
Never	167 (41.8%)	79 (39.5%)	88 (44.0%)	.46
Once in a while	80 (20.0%)	42 (21.0%)	38 (19.0%)	
Fairly often	91 (22.8%)	43 (21.5%)	48 (24.0%)	
Very often	62 (15.5%)	36 (18.0%)	26 (13.0%)	
“High” on drugs during sex with a FSW*				
Never	100 (25.0%)	51 (25.5%)	49 (24.5%)	.83
Once in a while	30 (7.5%)	17 (8.5%)	13 (6.5%)	
Fairly often	101 (25.3%)	51 (25.5%)	50 (25.0%)	
Very often	169 (42.3%)	81 (40.5%)	88 (44.0%)	
# Times had vaginal/anal sex with a FSW*	4.5 (2, 10)	4.5 (2, 9)	4.5 (2, 10)	.67
# Unprotected vaginal/anal sex acts with a FSW*	3.0 (2, 7)	3.0 (2, 6)	3.0 (2, 8)	.77
# Unprotected vaginal/anal sex acts with a spouse/steady partner*^a^	21.5 (8, 50)	20 (8, 52.5)	24 (8.5, 48)	.60
# Unprotected vaginal/anal sex acts with non-commercial casual partners*	4 (2, 10)	5 (2, 11.5)	4 (2, 10)	.73
***HIV/STI Services and Laboratory Results***				
Ever had an HIV test	180 (45.0%)	87 (43.5%)	93 (46.5%)	.55
Tested positive for any STI	31 (7.8%)	14 (7.0%)	17 (8.5%)	.58
Tested positive for Syphilis	12 (3.0%)	6 (3.0%)	6 (3.0%)	.80
Tested positive for Gonorrhea	2 (<1%)	1 (<1%)	1 (<1%)	1.00
Tested positive for Chlamydia	19 (4.8%)	8 (4.0%)	11 (5.5%)	.48

*Notes*: *past four months;

**Chi-square tests for categorical variables and t-tests for continuous variables.

^a^among those with a wife, steady partner, or girlfriend (n = 186).

Numbers associated with continuous variables represent medians and interquartile ranges.

### Sociodemographics

Median age was 37 years. A majority of the participants identified as Hispanic/Latino (87.5%). Approximately one-third (31.5%) of participants reported currently being married or in a common law marriage, and almost half (46.5%) reported having a wife, steady partner, or girlfriend. Median numbers of years of education was 9, and a majority (62.5%) reporting being employed at the time of the interview. Most (n = 356, 86.3%) identified as heterosexual, one participant reported being unsure about his sexual orientation, and the rest identified as bisexual (11.0%). Nineteen percent of participants reported spending any time in jail or a prison in the past four months, and 4.8% reported being currently on probation or parole.

### Alcohol and drug risks

Consistent with our pilot work with male clients of FSWs in Tijuana, most of the participants were active drug users, 93.3% of participants reported ever using any drug (marijuana, hash, heroin, inhalants, methamphetamine, ecstasy, cocaine, heroin and cocaine mixed together, and/or heroin and meth mixed together), and 80.3% reported using any drug in the past four months. Half (49.3%) of the sample screened positive for at least hazardous drinking according to the AUDIT.

### Sexual risks

In terms of sexual risk behavior, 87 (21.8%) and 127 (31.2%) participants reported being drunk or “high” on drugs during sex with a FSW in the past four months, respectively. In addition, participants reported having at least one unprotected sex act with a FSW, their spouse/steady partner, and/or a casual non-commercial partner during the last four months.

### HIV/STI Services and laboratory results

Almost half (45.0%) of the participants reported ever being tested for HIV. Approximately 8% of the sample tested positive for syphilis, gonorrhea, and/or chlamydia.

### San Diego vs. Tijuana participants

Table 2 reports differences between male clients from San Diego versus Tijuana. As expected, participants from San Diego were less likely to be Hispanic, and less likely to ever be deported from the U.S. compared to participants from Tijuana. Clients from San Diego were more likely to be born in the U.S., have more years of education, and to be employed. Also, clients from San Diego were less likely to be married or in a common law marriage, and were less likely to report residing with a spouse or steady partner than clients from San Diego. There were no differences between men from both cities in alcohol, drug, or sexual risk behaviors. However, San Diego participants were marginally more likely to report recent illicit drug use (84.8% vs. 75.9%).

**Table 2:** Descriptive statistics of Hombre Seguro participants by location: San Diego (n = 197) versus Tijuana (n = 203)

**Variable**	**Total (n = 400)**	**San Diego(n = 197)**	**Tijuana (n = 203)**	p**
***Sociodemographics***				
Age (years)	37 (30, 45)	38 (29, 45)	37 (30, 45)	.75
Hispanic (v. non-Hispanic)	350 (87.5%)	153 (77.7%)	197 (97.0%)	<.001
U.S.-born	82 (20.5%)	78 (39.6%)	4 (2.0%)	<.001
Ever deported from U.S.	136 (34.0%)	23 (11.7%)	113 (55.7%)	<.001
Married/common law	126 (31.5%)	44 (22.3%)	82 (40.4%)	<.001
Has a wife, steady partner, or girlfriend	186 (46.5%)	83 (42.1%)	103 (50.7%)	.08
Resides with spouse or steady partner	108 (27.0%)	41 (20.8%)	67 (33.0%)	.006
# of years of education	9 (7, 12)	11 (8, 12)	9 (6, 11)	<.001
Has children	276 (69.0%)	129 (65.5%)	147 (72.4%)	.13
Employed	250 (62.5%)	133 (67.5%)	117 (57.6%)	.04
Annual income	18 K (8 K, 30 K)	18 K (8.4 K, 30 K)		
Bisexual (v. Heterosexual)	44 (11.0%)	21 (10.7%)	23 (11.3%)	.83
Jailed*	77 (19.3%)	31 (15.7%)	46 (22.7%)	.08
Parole*	19 (4.8%)	13 (6.6%)	6 (3.0%)	.09
***Alcohol and Drug Risks***				
AUDIT score ≥ 8	197 (49.3%)	101 (51.3%)	96 (47.3%)	.43
Ever used any illicit drug	373 (93.3%)	187 (94.9%)	186 (91.6%)	.19
Recently used any illicit drug*	321 (80.3%)	167 (84.8%)	154 (75.9%)	.07
***Sexual Risks***				
Drunk during sex with a FSW*				
Never	167 (41.8%)	78 (39.6%)	89 (43.8%)	.81
Once in a while	80 (20.0%)	41 (20.8%)	39 (19.2%)	
Fairly often	91 (22.8%)	45 (22.8%)	46 (22.7%)	
Very often	62 (15.5%)	33 (16.8%)	29 (14.3%)	
“High” on drugs during sex with a FSW*				
Never	100 (25.0%)	40 (20.3%)	60 (29.6%)	.18
Once in a while	30 (7.5%)	15 (7.6%)	15 (7.4%)	
Fairly often	101 (25.3%)	51 (25.9%)	50 (24.6%)	
Very often	169 (42.3%)	91 (46.2%)	78 (38.4%)	
# Times had vaginal/anal sex with a FSW*	4.5 (2, 10)	6 (3, 11)	4 (2, 8)	.67
# Unprotected vaginal/anal sex acts with a FSW*	3.0 (2, 7)	3 (2, 8)	3 (2, 6)	.62
# Unprotected vaginal/anal sex acts with a spouse/steady partner*^a^	21.5 (8, 50)	30 (10, 60)	20 (7, 48)	.27
# Unprotected vaginal/anal sex acts with non-commercial casual partners*	4 (2, 10)	4 (2, 10)	5 (2, 12)	.96
***HIV/STI Services and Laboratory Results***				
Ever had an HIV test	180 (45.0%)	88 (44.7%)	92 (45.3%)	.90
Tested positive for any STI	31 (7.8%)	17 (8.6%)	14 (6.9%)	.52
Tested positive for Syphilis	12 (3.0%)	6 (3.0%)	6 (3.0%)	.20
Tested positive for Gonorrhea	2 (<1%)	1 (0.5%)	1 (0.5%)	.98
Tested positive for Chlamydia	19 (4.8%)	11 (5.6%)	8 (3.9%)	.44

Notes: *past four months;

**Chi-square tests for categorical variables and t-tests for continuous variables.

^a^among those with a wife, steady partner, or girlfriend (n = 186).

Numbers associated with continuous variables represent medians and interquartile ranges.

## Discussion

*Hombre Seguro* appears to be the first randomized controlled trial testing efficacy of an intervention to reduce sexual risk behaviors among male clients of FSWs. We successfully randomized participants to conditions, as there were no significant differences between clients in the *Hombre Seguro* and control conditions. We found predictable differences between clients who resided in San Diego versus Tijuana. However, we did find that interestingly, clients from San Diego were more likely to be single or living alone than clients from Tijuana, or conversely, married clients were more likely to reside in Tijuana, than San Diego. Given that the clients in this study were recruited based on sex with FSWs in Tijuana, this suggests that married male clients from Tijuana may perceive a more accessible and easier “escape” from wives or partners than married clients from San Diego. The opposite pattern may be found should a study be conducted with male clients of FSWs in San Diego, with married clients being more likely to reside in San Diego.

Male clients of FSWs represent an important bridge population. That is, male clients may bridge HIV/STI epidemics when they have unprotected sex with both higher-risk FSWs and their lower-risk wives or girlfriends. Male clients who reside in San Diego may also bridge epidemics in Tijuana, Mexico and the southwestern United States. Although San Diego and Tijuana clients did not differ in terms of sexual risk behavior or STIs, we did find a trend such that San Diego clients were more likely to have recently used illicit drugs than Tijuana clients. To the extent that these men are engaging in risky injection drug use (*i.e.*, needle sharing) with FSWs who inject drugs, or other people who inject drugs, in both cities, clients may be bridging drug use epidemics as well. In this situation, Hepatitis C viral transmission is also of great concern.

We maintained excellent follow-up rates in this study. This is attributed to our experience of working in the community, the fact that our local field staff were native to the language and community, and exceptional diligence of the research team in retaining participants.

The *Hombre Seguro* intervention was adapted from our previous research with FSWs in Tijuana and Ciudad Juarez, Mexico. We also used our pilot research with male clients in Tijuana to inform intervention development. Thus, we ensured that the intervention content was adapted and sensitive to the culture of our population. Researchers interested in adapting *Hombre Seguro* for male clients of FSWs in other parts of the world may model theoretical elements (e.g., motivational interviewing, role-playing to increase behavioral skills), while adapting to the cultural and contextual issues of the targeted population.

## Competing interests

The authors declare that they have no competing interests.

## Authors’ contributions

EVP participated in the design of the study and hypothesis development, performed the statistical analysis and drafted the manuscript. CVC participated in the coordination of the study and helped draft the manuscript. SJS participated in the design of the study and helped draft the manuscript. CM participated in the design of the study. SAS helped conceive of the study and participated in the design of the study. TLS conceived of the study, participated in the study design, and helped guide the statistical analysis. All authors read and approved the final manuscript.

## Authors’ information

Preparation of this manuscript was supported by a National Institute on Drug Abuse training fellowship (T32DA023356). This project was funded by a National Institute on Drug Abuse grant R01DA029008.

## Pre-publication history

The pre-publication history for this paper can be accessed here:

http://www.biomedcentral.com/1471-2458/14/475/prepub
